# Evaluation of the Gratitude Questionnaire in a Chinese Sample of Adults: Factorial Validity, Criterion-Related Validity, and Measurement Invariance Across Sex

**DOI:** 10.3389/fpsyg.2017.01498

**Published:** 2017-09-01

**Authors:** Feng Kong, Xuqun You, Jingjing Zhao

**Affiliations:** School of Psychology, Shaanxi Normal University Xi’an, China

**Keywords:** gratitude, confirmatory factor analysis, reliability, validity, measurement invariance

## Abstract

The Gratitude Questionnaire (GQ; McCullough et al., 2002) is one of the most widely used instruments to assess dispositional gratitude. The purpose of this study was to validate a Chinese version of the GQ by examining internal consistency, factor structure, convergent validity, and measurement invariance across sex. A total of 1151 Chinese adults were recruited to complete the GQ, Positive Affect and Negative Affect Scales, and Satisfaction with Life Scale. Confirmatory factor analysis indicated that the original unidimensional model fitted well, which is in accordance with the findings in Western populations. Furthermore, the GQ had satisfactory composite reliability and criterion-related validity with measures of life satisfaction and affective well-being. Evidence of configural, metric and scalar invariance across sex was obtained. Tests of the latent mean differences found females had higher latent mean scores than males. These findings suggest that the Chinese version of GQ is a reliable and valid tool for measuring dispositional gratitude and can generally be utilized across sex in the Chinese context.

## Introduction

With the emergence of positive psychology, gratitude has become more widely recognized in the literature. Gratitude has been often defined as a disposition, and reflects “a general tendency to recognize and respond with grateful emotion to the roles of other people’s benevolence in the positive experiences and outcomes that one obtains" (McCullough et al., 2002). In addition, gratitude has been also seen as a temporary emotion or mood state (Polak and McCullough, 2006). Numerous empirical studies have shown that gratitude is positively related to self-esteem, social support, life satisfaction and affective well-being, and negatively related to anger hostility and depressive symptoms (Emmons and McCullough, 2003; Wood et al., 2008, 2010; Hill et al., 2013; Kong et al., 2015b). This suggests gratitude is a crucial strength for achieving a good life. Therefore, it is important and necessary to study and measure gratitude.

To measure gratitude, researchers have developed several instruments. A first instrument is the 44-item Gratitude Resentment and Appreciation Test (GRAT) that measures gratitude as a disposition (Watkins et al., 2003). The GRAT assesses three aspects of gratitude: simple appreciation, lack of a sense of deprivation, and appreciation for others. The scale has demonstrated good psychometric properties (Watkins et al., 2003). A second instrument is the Gratitude Adjective Checklist (GAC) that measures gratitude as an emotion, mood, or disposition depending on the instructions (McCullough et al., 2002). The GAC is the sum of three adjectives—gratefulness, thankfulness, and appreciativeness. The GAC has demonstrated good psychometric properties (McCullough et al., 2002). A third instrument is the Gratitude Questionnaire (GQ) that measures dispositional gratitude (McCullough et al., 2002). The GQ including only several items can facilitate researchers to conduct a large-scale survey. Compared with the other two scales, the GQ has become the most widely used instrument for assessing dispositional gratitude in healthy populations, and is extensively used around the world. To our knowledge, the scale has been validated in many countries such as America, Italy, Chile, and Turkey (Froh et al., 2011; Yüksel and Oguz Duran, 2012; Jans-Beken et al., 2015; Caputo, 2016; Langer et al., 2016). Therefore, the aim of the present research was validate the scale in a Chinese population.

The GQ includes six items such as, “I feel thankful for what I have received in life,” and “I sometimes feel grateful for the smallest things.” In the study by McCullough et al. (2002), confirmatory factor analyses (CFA) showed that a robust one-factor structure of the GQ existed, which was replicated in many other studies (Froh et al., 2009; Yüksel and Oguz Duran, 2012; Caputo, 2016; Langer et al., 2016). Furthermore, McCullough et al. (2002) found that the GQ was positively related to life satisfaction, affective well-being and prosocial traits, and negatively related to psychological symptoms and materialism. Their results suggest that the GQ possesses good psychometric properties. Other researchers have also reported the criterion-related validity of the GQ. For example, Wood et al. (2007) found that undergraduate students with high gratitude reported higher life satisfaction and affective well-being. Furthermore, Lambert et al. (2009) found that gratitude predicted greater life satisfaction and less materialism among undergraduate students and life satisfaction mediated the relationship between gratitude and materialism. Taken together, these results further suggest that the GQ is a reliable and valid tool for measuring dispositional gratitude.

Subsequently, Leong (2009) developed a Chinese version of the GQ in Hong Kong using a standard back-translation procedure, and found that the reliability of the scale was high, ranging between α = 0.92–0.96. However, to our knowledge, they only reported the internal consistency reliability of the Chinese GQ, and did not systematically test its psychometric properties such as factorial validity, which is important and necessary. For example, Langer et al. (2016) found that item 6 (“Long amounts of time can go by before I feel grateful to something or someone”) was appropriate only in Chilean adults, rather than Chilean adolescents. Therefore, one aim of this study was to test the factorial validity and criterion-related validity in relation to subjective well-being of the Chinese version of the GQ in a large sample of Chinese adults.

Another important issue when using the GQ instrument is the measurement invariance across sex. Previous studies have compared the differences in the difference in the GQ scores between males and females (Kashdan et al., 2009; Sun and Kong, 2013; Kong et al., 2015b). For example, Kashdan et al. (2009) found that female adults scored higher on the GQ than male adults (Kashdan et al., 2009; Sun and Kong, 2013). In another study, Sun and Kong (2013) found that females had higher gratitude than males in late adolescence (Sun and Kong, 2013). However, when testing the sex difference in GQ scores, we have to assure that the GQ does measure the same latent structure across sex. That is, any sex difference in the GQ scores is due to real quantitative variations as opposed to measurement bias across male and females groups. Thus, another aim of this study was to examine whether the measurement structure underlying gratitude is equivalent across sex.

To address these questions, we firstly carried out a CFA to examine the structure of the Chinese GQ in a total of 1151 Chinese adults. We expected that the one-factor model fitted the data well. Second, we tested the criterion-related validity of the Chinese GQ in relation to life satisfaction and affective well-being. These two constructs were selected because of their reliable relationship with gratitude (McCullough et al., 2002; Emmons and McCullough, 2003; Wood et al., 2007; Toussaint and Friedman, 2009). We expected that gratitude would be significantly associated with life satisfaction and affective well-being. Thirdly, we carried out a multi-group CFA to test measurement invariance (i.e., configural, metric and scalar invariance) across sex. The establishment of measurement invariance across groups is a logical prerequisite to conducting substantive group comparisons (e.g., males and females).

## Materials and Methods

### Participants and Procedure

The study was conducted via an online survey website^1^. Participants are recruited through community forums. Before participation in the survey, written informed consent was obtained from each participant. A total of 1151 Chinese adults answered the questionnaires. In this sample, the mean age is 26.92 ± 5.59 years ranging from 17 to 57. More detailed demographic information is shown in **Table 1**. The study was approved by the institutional review board of Shaanxi Normal University.

**Table 1:** Characteristics of the present sample (*N* = 1151).

Variable	Level	Frequency	Rate (%)
Sex	Male	522	45.4
	Female	629	54.6
Education level	Senior school or below	49	4.3
	Bachelor	398	34.6
	Master	545	47.4
	Doctor or above	159	13.8
Age	20 or below	44	3.8
	21∼25	535	46.5
	26∼30	388	33.7
	31∼35	99	8.6
	36∼40	39	3.4
	40 or above	46	4.0

### Measures

To measure dispositional gratitude, we used the Chinese version of the GQ that was developed by Leong (2009) using a standard back-translation procedure^2^ and the Chinese GQ made slight adaptation in our study. For example, in our version, the word “appreciate” was translated into 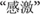, rather than 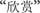. The GQ includes six items and each item is answered on a seven-point Likert scale ranging from 1 (strongly disagree) to 7 (strongly agree). Consistent with the original research of gratitude (McCullough et al., 2002), internal consistency was satisfactory with Cronbach’s α coefficient of 0.87.

To measure affective well-being, the 14-item Positive Affect and Negative Affect Scale (PANAS; Kuppens et al., 2008) was employed. Participants were asked to indicate how often they felt each effect in the past month on a seven-point Likert scale ranging from 1 = “not at all” to 7 = “almost or lasting.” The scale has good levels of reliability and validity in Chinese populations (Kuppens et al., 2008; Kong and Zhao, 2013). An affect balance score (positive affect minus negative affect score) was calculated as an index of affective well-being. In current study, the Cronbach’s α coefficient was 0.87 for positive affect and 0.85 for negative affect, respectively.

To measure life satisfaction, the Satisfaction with Life Scale (SWLS, Diener et al., 1985) was employed. This scale includes five items and each item is answered on a seven-point Likert type scale ranging from 1 (strongly disagree) to 7 (strongly agree). The SWLS has good levels of reliability and validity in Chinese populations (Kong et al., 2014, 2015a,c). In current study, the Cronbach’s α coefficient was 0.81.

### Data Analysis

To test whether the Chinese GQ has one-factor structure, a CFA was first performed using Amos 22.0. The Mardia’s coefficients for multivariate kurtosis were > 3, indicating significant multivariate non-normality in our data. Therefore, we used the Bollen-Stine bootstrap procedures (2,000 samples) with Maximum Likelihood (ML) estimation to accommodate the lack of multivariate normality. Because χ^2^ is sensitive to sample size, the following indices were used to evaluate the model’s goodness of fit: non-normal fit index (NNFI), comparative fit index (CFI), standardized root mean square residual (SRMR), and root mean square error of approximation (RMSEA). By convention, the fit was considered acceptable when NNFI and CFI ≥ 0.90, RMSEA ≤ 0.10, SRMR ≤ 0.05 (Hu and Bentler, 1999).

To evaluate measurement invariance (i.e., configural, metric, and scalar invariance) across sex, we conducted multi-group CFAs. Configural invariance refers to invariance of factor structure across sex groups. Metric invariance refers to invariance of factor loadings across sex groups. Scalar Invariance refers to invariance of item intercepts across sex groups. To evaluate differences of the models, we used the change for CFI and RMSEA (ΔCFI and ΔRMSEA) as indices (Gardner and Qualter, 2011; Martinez-Pecino and Durán, 2013). Strong invariance is supported when ΔCFI ≤ 0.01 and ΔRMSEA ≤ 0.015 (Cheung and Rensvold, 2002).

Finally, we tested sex-related latent mean differences. Tests of latent mean differences can provide more precise estimates regarding group differences than *t*-tests, because traditional *t*-tests ignore the impact of measurement error (Aiken et al., 1994). The z statistic was used to determine statistical significance between the latent means.

## Results

### Confirmatory Factor Analysis

**FIGURE 1:**
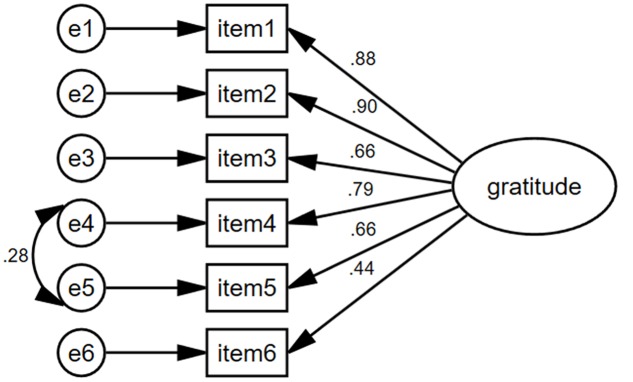
The structure model of the Gratitude Questionnaire.

The CFA analysis indicated that all the fit indices of the model, except RMSEA, meet their corresponding criteria, χ^2^(9) = 177.49, *p* < 0.001, NNFI = 0.92, CFI = 0.96, RMSEA = 0.13, SRMR = 0.04. Therefore, the proposed model provided an acceptable fit to the data. To improve the model, the modification indices were examined. Item 4 (“I am grateful to a wide variety of people”) and item 5 (As I get older I find myself more able to appreciate the people, events, and situations that have been part of my life history”) reflect gratitude span, so we allowed errors of these two items to correlate. The results found that the model showed a better fit to the data, χ^2^(8) = 103.14, *p* < 0.001, NNFI = 0.95, CFI = 0.97, RMSEA = 0.10, SRMR = 0.04. The factor loadings ranged from 0.44 to 0.90 (**Figure 1**).

### Measurement Invariance Across Sex

First, a multi-group CFA was carried out to test the configural invariance model in which the GQ has a one-factor structure across groups. The results showed that the model (M1) fitted very well, RMSEA = 0.075, CFI = 0.97, and all factor loading were significant (*p* < 0.001). These results demonstrate that the one-factor model fit the data well in both groups.

Second, following the configural invariance model, we tested a metric invariance model (M2) in which factor loadings are constrained to be equal across groups. The results showed the model fitted well, RMSEA = 0.065, CFI = 0.97. When compared to M1, there were no significant changes, ΔCFI = 0.001 and ΔRMSEA = 0.01 (**Table 2**). These findings suggest that factor loadings were invariant across the female and male groups.

**Table 2:** Fit indices for measurement invariance across sex.

Model	χ^2^	df	CFI	RMSEA	Comparison	Δχ^2^	ΔCFI	ΔRMSEA
M1	119.57	16	0.97	0.075				
M2	121.96	21	0.97	0.065	M2 vs. M1	2.39	0.001	0.01
M3	133.59	27	0.97	0.059	M3 vs. M2	11.63	0.001	0.006

*CFI, comparative fit index; RMSEA, root mean square error of approximation; M1, configural model; M2, metric model; M3, scalar model. In every case theΔχ^*2*^ was not significant (*ps* > 0.05).*

Finally, we tested a scalar invariance model (M3) in which intercepts and factor loadings are constrained to be equal across the female and male groups. The results showed the model fitted well, RMSEA = 0.059, CFI = 0.97. When compared to M2, there were no significant changes, ΔCFI = 0.001 and ΔRMSEA = 0.006 (**Table 2**). These findings suggest that intercepts were invariant across the female and male groups.

Taking together, these results suggest that measurement invariance of the GQ holds across sex.

### Testing for Latent Mean Differences

To estimate latent mean differences between different sex groups, we carried out a multi-group CFA. The male group was selected as a reference group and its latent mean is fixed to zero. Latent mean for the female group is freely estimated. The results revealed that females had higher scores than males in the GQ (*Z* = 2.39, *p* = 0.02).

### Reliability

The internal consistency of the model was assessed by computing the composite reliability (Raykov, 1997). In comparison with Cronbach’s alpha reliability, composite reliability does not assume that all indicators are equally reliable. If the composite reliability is more than 0.7, the model is satisfactory. In the total sample, the composite reliability for the scale was 0.87. In males the composite reliability for the scale was 0.86, and in females the composite reliability for the scale was 0.88, indicating that the internal consistency of the model is satisfactory.

### Criterion-Related Validity

Because our data were collected at one time from one source, common-method bias might occur. We applied Harman’s single-factor test to examine common method bias. A model with one factor (with all items for the three scales loading on a unique factor) was built. The results revealed that the model did not fit the data well [χ^2^_(275)_ = 7814.55, *p* < 0.001, CFI = 0.55, GFI = 0.49, SRMR = 0.13, RMSEA = 0.15]. According to these results, we believe that common method bias might not be a serious issue in the current study.

To verify the criterion-related validity of the Chinese GQ, regression analyses were conducted with the total sample to test its relation with life satisfaction and affective well-being. The result revealed that gratitude explained 5.1% of the variance in life satisfaction (β = 0.23, *p* < 0.001) and explained 3.8% of the variance in affective well-being (β = 0.19, *p* < 0.001).

Because the significant sex difference in gratitude was found in the previous analysis, we wondered whether there also exists the sex difference in the relation of gratitude with life satisfaction and affective well-being. Therefore, we conducted an exploratory analysis to test whether sex moderates the influence of gratitude on life satisfaction and affective well-being. The results found no significant interaction effect for life satisfaction (β = -0.12, *p* > 0.05) and affective well-being (β = -0.06, *p* > 0.05). This indicated that sex did not moderate the influence of gratitude on life satisfaction and affective well-being.

## Discussion

The current study aimed to examine the validity of the Chinese version of GQ and measurement invariance across sex. As with the original English version of the GQ (McCullough et al., 2002), the Chinese GQ showed satisfactory internal consistency reliability. Moreover, consistent with the English GQ (McCullough et al., 2002), the CFA yielded a one-factor structure, with an adequate model fit. Besides, the multi-group CFA analysis demonstrated that the one-factor structure held across sex. These results suggest that the Chinese version of the GQ is a reliable and valid measure of dispositional gratitude.

Importantly, our study extended previous studies by examining the sex invariance of the Chinese GQ. In current study, the configural, metric and scalar invariance of the Chinese GQ held across sex groups, indicating that the Chinese GQ measures the same construct for different sex groups. To our knowledge, this is the first study to demonstrate the factor invariance across sex of the Chinese GQ. In addition, consistent with findings reported previously in traditional mean differences (Kashdan et al., 2009; Sun and Kong, 2013), tests of the latent mean differences found females had higher latent mean scores than males. Therefore, the pattern of sex differences is not likely explained by measurement bias, but rather can be interpreted as real quantitative variations that arise from psychological influences. One possible explanation for the sex differences is that females tend to have greater willingness to express emotions and obtain more benefit from it (Kashdan et al., 2009).

For the criterion-related validity, we tested the association between gratitude and well-being indicators including life satisfaction and affective well-being. The Chinese GQ could be significantly associated with life satisfaction and affective well-being, which is similar with the findings in previous studies (McCullough et al., 2002; Emmons and McCullough, 2003; Wood et al., 2007; Toussaint and Friedman, 2009). These results suggest that gratitude is an important character strength for achieving a happy life. Furthermore, we found that sex did not moderate the relationship between gratitude and life satisfaction, as well as the relationship between gratitude and affective well-being in Chinese adults. This is consistent with the findings by Froh et al. (2009). They found that sex did not moderate the effects of gratitude on life satisfaction and affective well-being in early adolescence. Together with our findings, these results suggest that, from adolescence to adulthood, sex does not influence the association between gratitude on subjective well-being. Given that there are sex differences in scores on the GQ, further studies should test whether there are different underlying mechanisms of how gratitude influences subjective well-being.

### Strengths and Limitations

To sum up, this study adds to the existing literature on gratitude in several ways. First, the Chinese GQ had good internal consistency (e.g., composite reliability) and factorial validity, suggesting that researchers can use the GQ to measure gratitude in Chinese adults. Second, the Chinese GQ had good criterion-related validity with measures of life satisfaction and affective well-being and sex did not moderate the effects of gratitude on these two constructs, indicating the males and females with higher gratitude tend to experience a happier life. Third, the Chinese GQ had configural, metric and scalar invariance across male and female groups, which suggests gratitude researchers don’t have to consider the sex matter when measuring gratitude. In addition, through tests of the latent mean differences, females had higher latent mean scores than males. The method considers the influence of measurement errors, so it can reflect the real sex differences.

Despite of these strengths, three limitations need to be considered. First, study participants were adults above 17 years old, and findings cannot be generalized to teenagers. Second, we only tested the sex invariance; therefore, further studies should test measurement invariance across other groups such as age groups. Finally, only the convergent validity of the GQ was tested, so further studies should also test its discriminant validity with other variables.

## Ethics Statement

The study was conducted according to the Declaration of Helsinki and was approved by the Shaanxi Normal University committee.

## Author Contributions

All authors listed have made a substantial, direct and intellectual contribution to the work, and approved it for publication.

## Conflict of Interest Statement

The authors declare that the research was conducted in the absence of any commercial or financial relationships that could be construed as a potential conflict of interest.

## Footnotes

1 www.sojump.com

2 http://www.psy.miami.edu/faculty/mmccullough/Gratitude-Related%20Stuff/Chinese%20GQ-6_Joyce%20Leong.pdf
